# Decreased step count prior to the first visit for MDD treatment: a retrospective, observational, longitudinal cohort study of continuously measured walking activity obtained from smartphones

**DOI:** 10.3389/fpubh.2023.1190464

**Published:** 2023-09-28

**Authors:** Yoshihisa Fujino, Fumie Tokuda, Shinji Fujimoto

**Affiliations:** ^1^University of Occupational and Environmental Health Japan, Fukuoka, Japan; ^2^Japan Medical Office, Takeda Pharmaceutical Company Limited, Tokyo, Japan

**Keywords:** continuous monitoring, diagnosis, Japanese, major depressive disorder, physical activity, step count, antidepressant

## Abstract

**Introduction:**

Major depressive disorder (MDD) is a common debilitating psychiatric condition and a major cause of productivity loss in workers. Using intermittent, subjective indicators, previous studies have shown that physical activity can predict lower levels of depressive symptoms. However, there is an unmet need for continuous and objective measures to identify MDD development before it results in productivity loss. The aim of this study was to elucidate the association between continuously measured walking activity and the development of MDD.

**Methods:**

This retrospective, observational, longitudinal cohort study used health insurance claims data. Individuals aged 20–74 years were included if they had a record of MDD diagnosis and daily step count data for the 60 days before and after the first recorded MDD-related visit, which was defined as the index date. Multivariate analysis was conducted to compare 7-day moving averages of step counts on each day of the analysis period with the mean step count on the index date. Joinpoint regression analysis was used to determine when the trajectory of the moving step count average changed (inflection point).

**Results:**

In total, 2,143 patients with a mean age of 41.2 (standard deviation [SD]: 10.6) years were included. The majority of patients were men (69.5%) and employed full-time (94.1%). Antidepressants were prescribed for 59.2% of patients. The 7-day moving average step count decreased from 6,310 (SD: 3758) at day −60 to 5,879 (SD: 3183) at the index date (first recorded MDD-related visit), and then increased to 6,062 (SD: 4029) at day +60. Compared with the index date, the 7-day moving average of step counts was significantly higher at days −60 to −1, +23 to +33, and + 42 to +60, and significantly lower at days +2 and + 3. Joinpoint regression analysis of 7-day moving average step counts from day −60 to day 0 identified an inflection point at day −13.

**Conclusion:**

In working-age Japanese people, a formal diagnosis of MDD was preceded by a notable decline in daily step counts by approximately 2 weeks. MDD diagnosis and (presumed) treatment were followed by a gradual increase in daily step counts.

## Introduction

The number of people with mental health disorders in Japan continues to increase (1). Major depressive disorder (MDD) is a common and debilitating psychiatric condition. In Japan, the 12-month prevalence estimates for MDD are 2.2% in men and 3.2% in women (2). MDD is characterized by a variety of emotional and physical problems, and is often associated with impaired psychosocial functioning, which typically manifests in productivity loss (3, 4). The overall economic burden associated with MDD in Japan was estimated at approximately $11 billion per year in 2008, of which approximately $7 billion was associated with productivity loss due to presenteeism and absenteeism (5). The proportion of companies in Japan with employees who retired or took leave that lasted more than 1 month owing to mental health issues was 9.2% in 2020, down only slightly from 10.3% in 2013 (1). Mental health disorders are the most common cause of medically certified sick leave lasting 30 days or longer, accounting for 52% of such absences in men and 35% in women (6).

In Japan, a range of measures have been implemented to combat depression among employees, such as discouragement of long working hours, mental health care in the workplace and the Stress Check Program (7–9). Long working hours, traditionally part of Japanese workplace practice, are one of the factors contributing to work-related accidents caused by mental health issues (8, 9). As a result, the practice of working long hours is being discouraged (10). The Stress Check Program has been mandated by law since 2015 for companies with 50 or more employees (7, 11). As part of the Program, employers are required to provide workers with a psychosocial stress questionnaire at least once a year. If the results of the questionnaire suggest that a worker has high stress levels, employers will arrange (if requested by the worker) an interview with an occupational health professional. Employers are required to consider the opinion of the occupational health professional on how the working conditions of employees with high stress may be improved and are prohibited from taking retaliatory actions against such employees (7, 11). In addition to the Stress Check Program, four types of care have been recommended for employees with mental health issues in the workplace: the promotion of mental health awareness among workers, promotion of awareness and response to mental health issues by supervisors (line care), care by occupational health professionals, and care by outside specialists including employee assistance program counselors (12).

As the number of people with mental health disorders continues to increase, the importance of proactively identifying at-risk workers is increasing. Stress checks and line care are aimed at early detection of mental health problems in workers (7, 11). However, stress checks are based on self-reporting and self-assessment by the workers themselves, and line care relies on the subjective judgment of supervisors (7, 11). Therefore, there is an unmet need for continuous and objective methods and techniques to identify mental health problems in workers in a timely manner, before they result in aggravation of symptoms along with productivity loss. However, no such methods have been established so far.

Previous studies have established the connection between physical activity and depressive symptoms (13–16). Evidence from a systematic review of 49 prospective cohort studies (*N* = 266,939 individuals, 1,837,794 person–years) conducted across Asia (including Japan), Europe, North America, and Oceania, found that higher physical activity has protective effects against the development of MDD and depressive symptoms regardless of age or geographic location (16). Indeed, physical activity has a direct antidepressant effect, which is mediated via several physiological and psychosocial pathways (15).

However, in previous studies, physical activity was measured intermittently using a self-reported questionnaire or a pedometer (13, 14, 16). It may be difficult to detect the signs and symptoms of MDD using intermittent, subjective indicators. More recently, smartphones and wearable devices with accelerometer and gyroscope sensors have been developed and are available for the public. Most of these devices are equipped with telecommunication functions that enable automatic collection of real-time data. Therefore, an objective marker that can be continuously monitored using a wearable device may be useful in detecting the signs of MDD prior to a clinical diagnosis. To the best of our knowledge, no study has assessed physical activity before and after MDD diagnosis using objective measures obtained via continuous monitoring.

The present study was conducted to elucidate the association between continuously measured walking activity and the development of MDD. We hypothesized that the diagnosis of MDD would be preceded by a reduction in daily step counts and followed by a recovery of daily step counts due to interventions. If an association between walking activity and MDD development was found, we sought to identify the number of days prior to the diagnosis that this decrease would occur.

## Materials and methods

### Study design and participants

**Figure 1:**
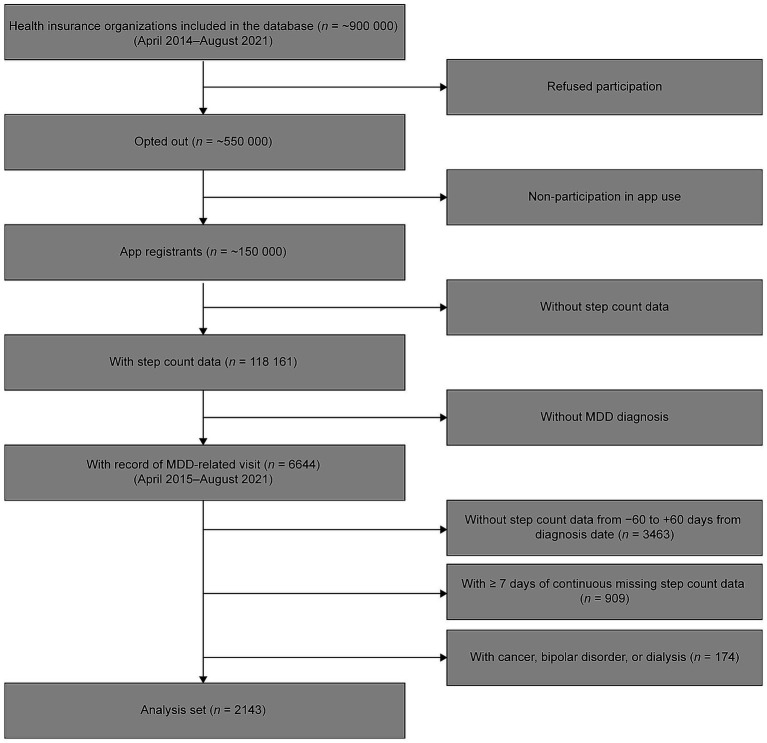
Patients included in the analysis. MDD, major depressive disorder.

This was a retrospective, observational, longitudinal cohort study. We used longitudinal data on health insurance claims and routine health examinations from over 550,000 people enrolled in employment insurance at approximately 80 health insurance associations located across various regions of Japan. In addition, daily step count data were collected from over 150,000 of the insured individuals and their dependents who are enrolled in health service apps provided by the health insurance associations. Of these approximately 150,000 individuals, both the health insurance claims and step count data for the period between April 1, 2014 and August 31, 2021 were obtained for 118,161 individuals. Of this number, 6644 individuals were aged 20–74 years and had a record of MDD diagnosis [International Classification of Diseases (ICD) 10 codes F32–F33]. After excluding those without step count data for the 60 days before and after the date of the first recorded MDD-related visit (*n* = 3,463), those with missing step count data on 7 or more consecutive days (*n* = 909), and those with records of cancer (ICD-10 codes C00–C97), bipolar disorder (F31) or dialysis (identified by searching for the term ‘dialysis’ in Japanese in medical procedure records) (*n* = 174), data from 2,143 patients were available for the present analysis (Figure 1).

### Observation and analysis period

Health insurance claims data and step count data for the period between April 2014 and August 2021 and MDD diagnosis data for the period between April 2015 and August 2021 were obtained and analyzed in the present study. For each included individual (*n* = 2,143), the index date was defined as the date of the first recorded MDD-related visit identified in the health insurance claims data. Step count data were analyzed over a period spanning 60 days before and 60 days after the index date (analysis period), to allow for changes in walking activity to be detected based on the diagnostic criteria for MDD (several symptoms during the same 2-week period) with sufficient sample size.

### Assessment of step count

Daily step count data, including the year, month and day when step count data were acquired, were recorded and collected via the Health app (iOS) or Google Fit app (Android), which rely on the smartphone’s inbuilt accelerometer. Data on participation in walking campaigns (assessed daily) were also collected via health service apps. Walking campaigns are events in which health insurance associations invite participants to engage in organized walking through the health service apps to promote physical activity in the participants’ daily lives. If data were available on the start and end dates of the campaign, all days between the start and end dates were considered as participation days. These data (year and month when step count data were acquired, participation in walking campaigns) were included in a multivariate regression analysis model as covariates (see below).

### Other covariates

Data on age, sex, comorbidity, hospitalization events and antidepressant prescriptions were also collected, and were derived from health insurance claims data. Age was calculated by comparing the birth month and year with the month and year of the index date, defined as the date of the first recorded MDD-related visit or, in sensitivity analysis, as the date of the first prescription of antidepressants. The presence of comorbidity (assessed monthly) was defined as having one of the following diseases in the same year, month or week as the step count time window: coronary artery disease (ICD-10 codes I20–I25), diabetes (E10–E14), stroke (I60–I63), chronic lower respiratory tract diseases (J40–J47), arthritis (M00–M13), hypertension (I10–I15), osteoporosis (M80–M82) and fractures (S02, S12, S22, S32, S42, S52, S62, S72, S82, S92, T02). Hospitalization events (assessed daily) were identified based on hospitalization costs and were not limited to a specific disease. Prescription of antidepressants was defined as the prescription of any of the following drugs during the analysis period: amitriptyline hydrochloride, amoxapine, clomipramine hydrochloride, dosulepin hydrochloride, duloxetine hydrochloride, escitalopram oxalate, fluvoxamine maleate, imipramine hydrochloride, lofepramine hydrochloride, maprotiline hydrochloride, mianserin hydrochloride, milnacipran hydrochloride, mirtazapine, nortriptyline hydrochloride, paroxetine hydrochloride hydrate, sertraline hydrochloride, setiptiline maleate, trazodone hydrochloride, trimipramine maleate, venlafaxine hydrochloride and vortioxetine hydrobromide. These data (age, sex, comorbidity, hospitalization events and antidepressant prescriptions) were also used as covariates (see below).

### Statistical analysis

Demographic and clinical characteristics of included patients were analyzed using summary statistics. For continuous variables, means and standard deviations (SDs) or medians and interquartile ranges were calculated depending on the data distribution. For categorical variables, frequencies and/or percentages were calculated. An alpha level of 0.05 was used when testing for statistical significance.

The 7-day moving average of step counts was calculated for each day of the analysis period. Step counts on the index date were excluded from the calculation, as were step counts on days when fewer than 50 steps were recorded, on the assumption that fewer than 50 steps would indicate that the individual was not carrying a smartphone on that day. Missing data were not imputed. Days for which step count data were not available were excluded from the moving average calculation.

Multivariate regression analysis was conducted using generalized estimating equations (GEEs) to compare 7-day moving averages of step counts on each day with the mean step count on the index date. For GEEs, a binomial distribution (log link) was specified as a fixed value for the error structure, and the Quasi-Likelihood Information Criterion (QIC) selected autoregressive (smallest QIC score) for the correlation structure. Covariates (age, sex, comorbidity, year and month when step count data were acquired, participation in walking campaigns, hospitalization events and antidepressant prescriptions) were included in the model to correct for potential confounding. The duration of participation in walking campaigns was included as a covariate, because the number of steps was likely to be affected by participation in such campaigns.

Joinpoint regression analysis was used to determine when the trajectory of the moving step count average changed. In a joinpoint regression analysis, lines representing data trends on a graph are converted into a series of straight lines linked at joinpoints (17). The minimum and maximum number of joinpoints are provided by researchers. Analysis begins with the minimum number of joinpoints specified. The model is then built by gradually increasing the number of joinpoints, until the maximum specified number is reached. How well each model fits the data is compared using the Monte Carlo Permutation method, and the model with the best fit is selected. The location of joinpoints corresponds to statistically significant changes in the trajectory of the data trend (inflection points) (17). In the present study, the minimum and maximum number of joinpoints were set at 0 and 1, respectively. If the model containing 1 joinpoint fits the data better than the model containing 0 joinpoints, then the location of the joinpoint would be considered the inflection point.

In addition to the main analysis, subgroup analyses were conducted according to age at the index date (< 40 vs. ≥ 40 years old), sex (male vs. female) and type of health insurance plan (own plan vs. on a family member’s plan as a dependent). The effects of age and sex on the level of physical activity are well established (18). Subgroup analysis by type of health insurance plan was conducted because we hypothesized that individuals on their own plan would be more likely to have an active social life and, as a result, take more steps until immediately before the index date compared with individuals on a family member’s plan. This would be reflected in a greater change in the number of steps around the index date in participants on their own plan than in participants on a family member’s plan. Furthermore, a sensitivity analysis using the first prescription of antidepressants as the index date was performed.

Analyses were performed using SAS software, version 9.4 (SAS Institute, Cary, North Carolina, United States). Joinpoint regression analysis was performed using Joinpoint Regression Program, version 4.9.1.0 (US National Institutes of Health, Bethesda, Maryland, United States).

### Compliance with ethics guidelines

This study was based on anonymized administrative claims data that did not involve patients directly. Thus, ethics approval and informed consent were not required, per the Ethical Guidelines for Medical Research Involving Human Subjects issued by the Japanese Ministry of Health, Labor and Welfare.

## Results

### Patient characteristics

Among the 2,143 patients included in the present study, the proportion of men (69.5%) was higher than the proportion of women (30.5%) (Table 1). The mean age of included patients was 41.2 (SD: 10.6) years. Most patients were on their own health insurance plan (94.1%) and over half (59.2%) were prescribed antidepressants during the analysis period. The most common comorbidities were hypertension (12.1%), chronic lower respiratory disease (11.6%) and diabetes mellitus (6.7%). Few patients had conditions that could have affected their step counts, such as bone fractures, osteoporosis or arthritis (< 3% per condition) (Table 1).

**Table 1:** Characteristics of included patients.

Characteristic	*N* = 2,143
Age, mean ± SD (range)	41.2 ± 10.6 (18–70)
< 40 years, *n* (%)	905 (42.2)
≥ 40 years, *n* (%)	1,238 (57.8)
Men, *n* (%)	1,489 (69.5)
CCI, mean ± SD (range)	0.5 ± 0.8 (0–8)
Health insurance type, *n* (%)	
Own plan	2017 (94.1)
Dependent family member	126 (5.9)
Patients with events during the analysis period, *n* (%)	
Walking campaign	244 (11.4)
Hospitalization	67 (3.1)
Prescription of antidepressants^a^	1,268 (59.2)
Comorbidities, *n* (%)	
Coronary artery diseases	54 (2.5)
Diabetes	144 (6.7)
Stroke	22 (1.0)
Chronic lower respiratory diseases	249 (11.6)
Arthritis	57 (2.7)
Hypertension	259 (12.1)
Osteoporosis	22 (1.0)
Fracture	21 (1.0)

^a^Assessed during the analysis period.

CCI, Charlson comorbidity index; SD, standard deviation.

### Step counts before and after the index date (main analysis)

**Figure 2:**
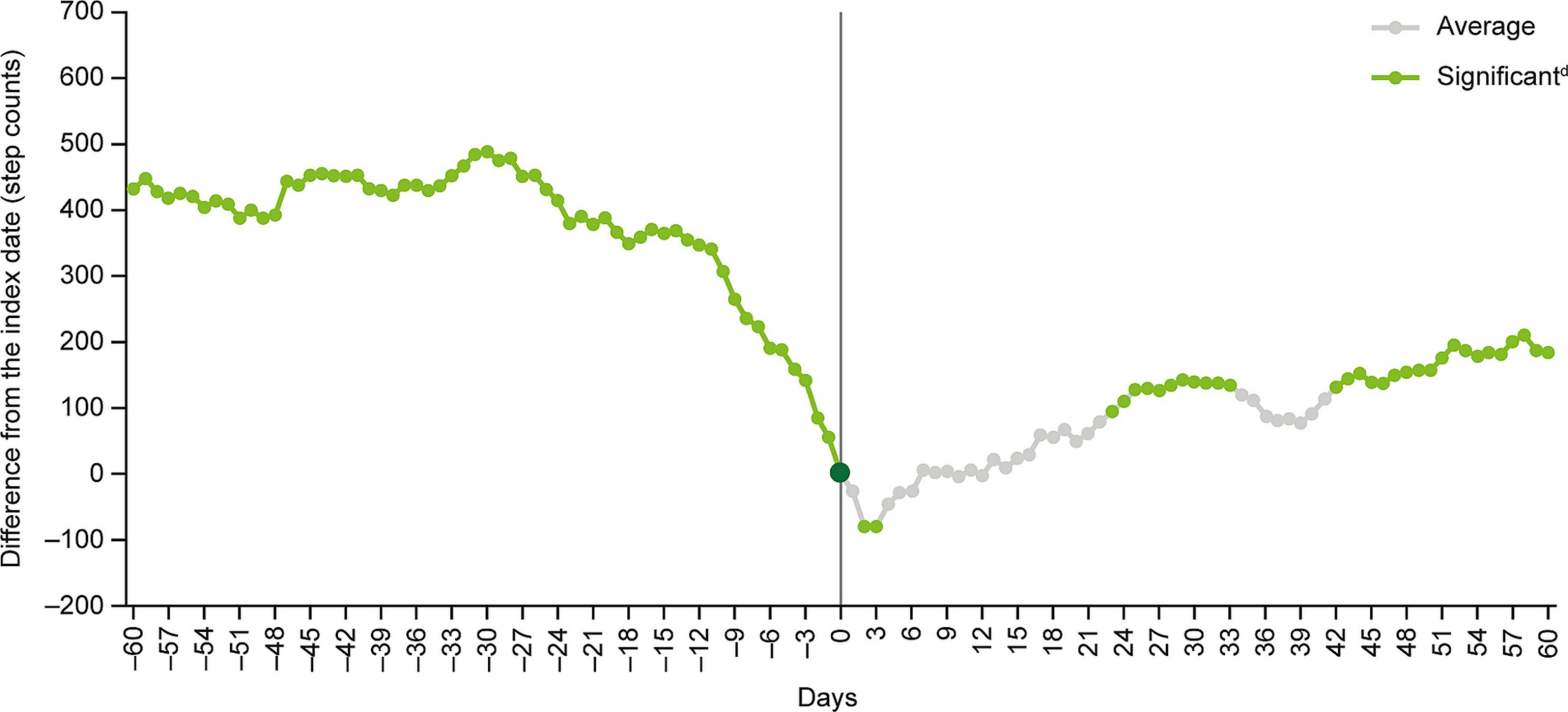
Multivariate analysis^a^ of moving average step counts^b^ before and after the index date^c^. ^a^Covariates were age, sex, year and month when step count data were acquired, participation in a walking campaign, hospitalization event, prescription of antidepressants during the analysis period and presence of comorbidity (coronary artery diseases, diabetes, stroke, chronic lower respiratory diseases, arthritis, hypertension, osteoporosis, fracture). ^b^The moving average of step counts was calculated after subtracting the number of steps on day 0 (i.e., the index date) from the 7-day moving average for each day at the individual level. ^c^The index date (day 0) was defined as the date of the first recorded MDD-related visit identified from health insurance claims data. ^d^Step count changes around the index date were examined by inspecting the statistical significance of regression coefficients corresponding to each time point. MDD, major depressive disorder.

A notable decline in mean daily step counts occurred in the 2 weeks before the index date. After the index date, mean daily step counts increased slowly, with differences versus the index date becoming significant after approximately 1 month (Figure 2).

The 7-day moving average step count decreased from 6,310 (SD: 3758) at day −60 to 5,879 (SD: 3183) at the index date, and then increased to 6,062 (SD: 4029) at day +60 (Table 2). The index date-adjusted moving average step count was 431.5 (SD: 3148) at day −60, decreasing to negative values at days +1 to +6, +10 and + 12, before increasing to 183.6 (SD: 3570) at day +60 (Table 2 and Figure 2).

**Table 2:** Daily step counts during the analysis period.

Day^a^	*n*	Moving mean^b^	SD	Median	Difference from index date^c^			Multivariate analysis^d^		
					Moving mean^b^	SD	Median	Estimates	95% CI	*p* value
−60	2,143	6,310	3,758	5,903	432	3,148	248	1.07	1.04–1.09	0.000
−50	2,143	6,278	3,367	5,944	400	2,729	291	1.06	1.04–1.08	0.000
−40	2,143	6,310	3,230	6,039	432	2,607	311	1.07	1.05–1.09	0.000
−30	2,143	6,367	3,245	6,025	488	2,621	317	1.08	1.06–1.10	0.000
−20	2,143	6,267	3,132	5,899	389	2,503	209	1.06	1.05–1.08	0.000
−15	2,143	6,242	3,073	5,971	364	2,419	250	1.06	1.04–1.08	0.000
−10	2,143	6,184	3,152	5,857	306	2,367	221	1.05	1.04–1.07	0.000
−5	2,143	6,067	3,065	5,772	188	1789	118	1.03	1.02–1.05	0.000
−1	2,143	5,935	3,175	5,740	56	738	11	1.01	1.00–1.02	0.000
0^e^	2,143	5,879	3,183	5,596	Reference			Reference		
1	2,143	5,853	3,228	5,462	−26	777	−8	1.00	0.99–1.00	0.122
10	2,143	5,874	32,435	5,590	−4	2,293	15	1.00	0.98–1.02	0.994
20	2,143	5,928	3,193	5,591	49	2,535	49	1.01	0.99–1.03	0.274
30	2,143	6,018	3,675	5,631	140	2,959	62	1.03	1.00–1.05	0.017
40	2,143	5,969	3,584	5,535	90	2,943	47	1.02	1.00–1.04	0.101
50	2,143	6,035	3,904	5,595	157	3,318	46	1.03	1.00–1.05	0.018
60	2,143	6,062	4,029	5,700	184	3,570	70	1.04	1.01–1.06	0.006

^a^Daily data were used in the analysis, however, only the results for every 10 days are shown here.

^b^7-day moving mean.

^c^The mean and median were calculated after subtracting the number of steps on day 0 from the 7-day moving average for each day at the individual level.

^d^Covariates were age, sex, year and month when step count data were acquired, participation in a walking campaign, hospitalization event, prescription of antidepressants during the analysis period and presence of comorbidity (coronary artery diseases, diabetes, stroke, chronic lower respiratory diseases, arthritis, hypertension, osteoporosis, fracture).

^e^The index date was defined as the date of the first recorded MDD-related visit identified from health insurance claims data.

CI, confidence interval; SD, standard deviation.

The results of the multivariate GEE model analysis showed that, compared with the index date, the 7-day moving average step counts were significantly higher at days −60 to −1, +23 to +33 and + 42 to +60, and significantly lower at days +2 and + 3 (Figure 2).

**Figure 3:**
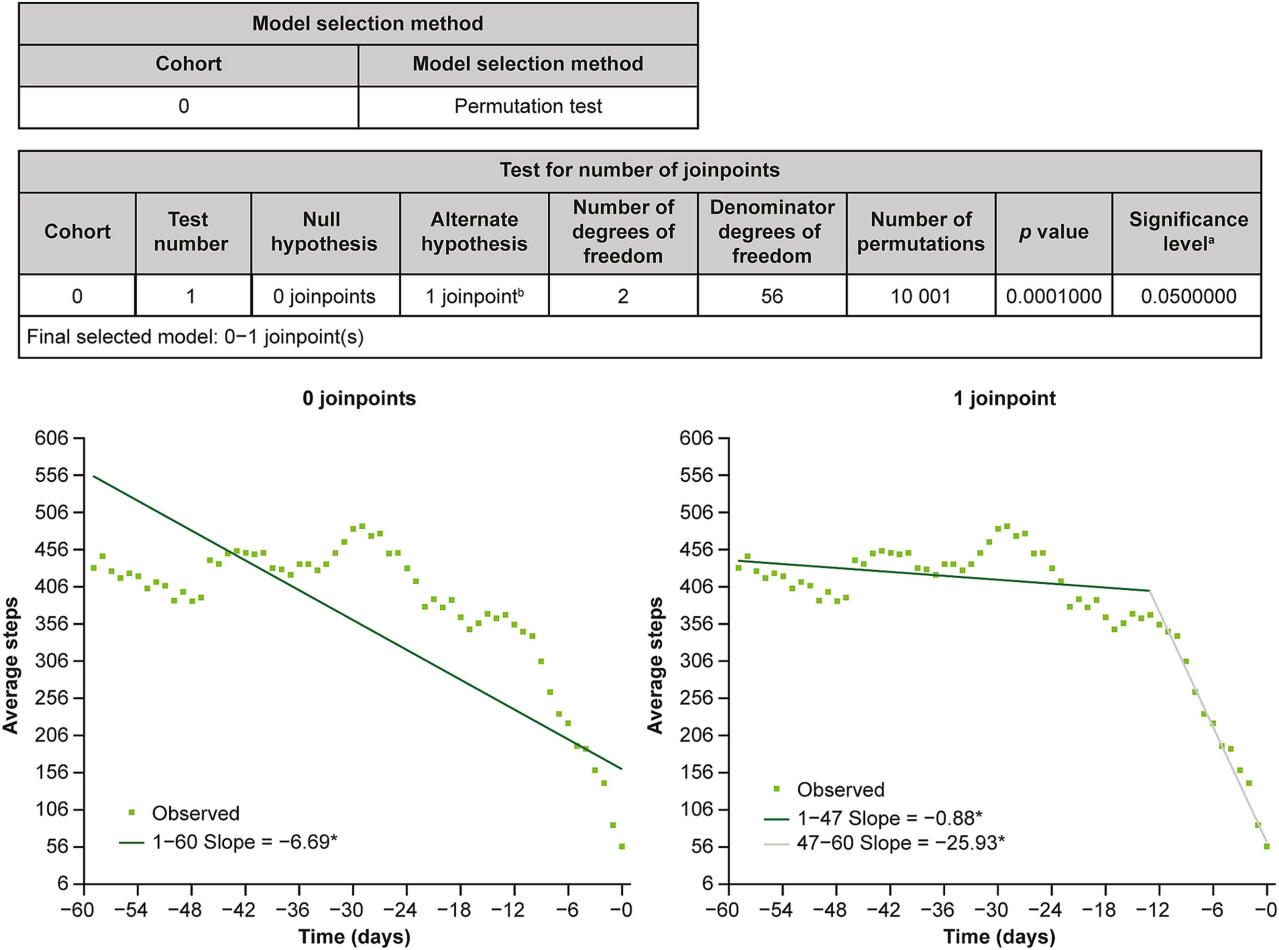
Results of joinpoint regression analysis. ^a^Significance level for individual test. ^b^Final selected model. *The slope is significantly different from 0 at the alpha level of 0.05.

Joinpoint regression analysis of 7-day moving average step counts from day −60 to day 0 identified an inflection point at day −13 (Figure 3).

**Figure 4:**
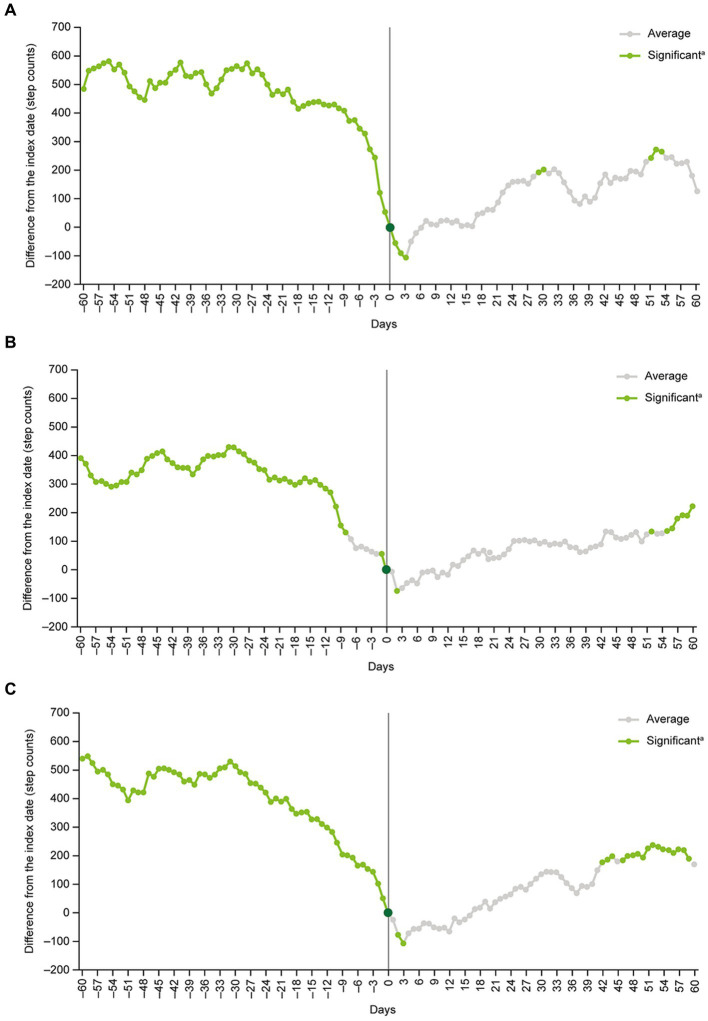

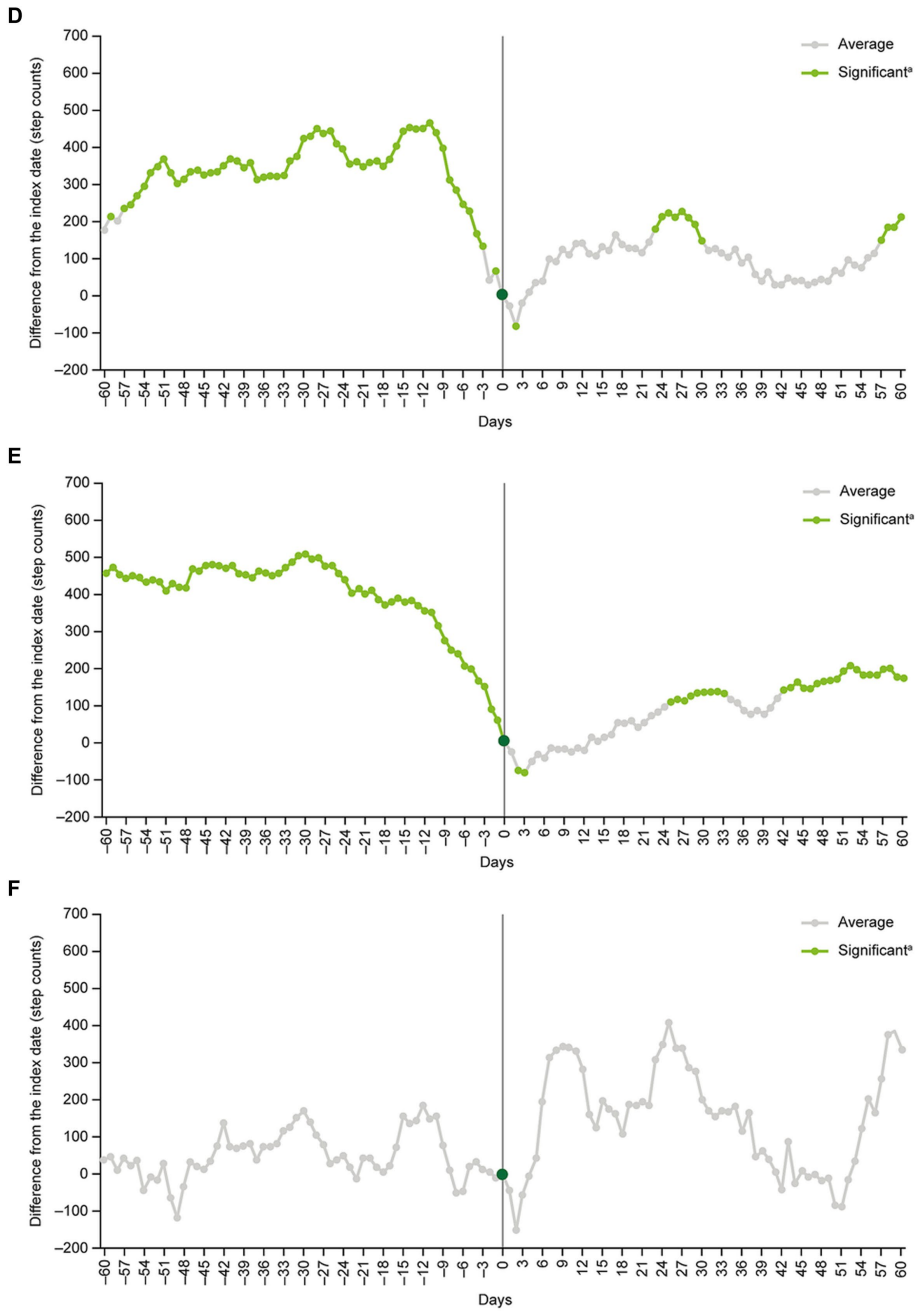
Subgroup analyses in **(A)** patients <40 years old, **(B)** patients ≥40 years old, **(C)** men, **(D)** women, **(E)** patients on their own health insurance plan, and **(F)** patients on a family member’s health insurance plan as a dependent. ^a^Step count changes around the index date were examined by inspecting the statistical significance of regression coefficients corresponding to each time point.

Subgroup analyses of patients aged <40 years versus ≥40 years, men versus women, and patients on their own health insurance plan versus those on a family member’s plan as a dependent generally produced similar findings to the main analysis (Figure 4). The exception was the subgroup of patients who were on a family member’s healthcare plan as a dependent (*n* = 126). In this subgroup, no significant differences in daily step counts compared with the index date were detected at any point during the analysis period (Figure 4).

### Step counts before and after first antidepressant use (sensitivity analysis)

Using the date of the first antidepressant prescription (instead of the date of the first record of MDD diagnosis) as the index date identified a total of 2022 patients who were included in the sensitivity analysis. Patients’ characteristics, the overall trajectory of mean step counts and results of the multivariate GEE model analysis in the sensitivity analysis were similar to those in the main analysis (data on file).

### Effects of model covariates on step counts

Of the covariates included in the multivariate model assessing step counts before and after the index date (main analysis; *N* = 2,143), age 40 years or older (*Z* = −2.39, *p* = 0.017), coronary artery disease (*Z* = −2.80, *p* = 0.005), arthritis (*Z* = −2.57, *p* = 0.010), hospitalization (*Z* = −6.36, *p* = 0.000) and prescription of antidepressants (*Z* = −3.11, *p* = 0.002) were independently associated with lower mean step counts during the study period, whereas male sex (*Z* = 11.76, *p* = 0.000) was associated with higher mean step counts. Mean step counts were also significantly higher in March (*Z* = 2.01, *p* = 0.044) and April (*Z* = 2.87, *p* = 0.004) versus December, and in 2015–2020 (*Z* = 2.99–4.07, *p* = 0.000–0.003) versus 2021.

## Discussion

This study examined the relationship between MDD and physical activity in the form of daily step counts. The results show a sharp decline in daily step counts during the 13 days before the patients’ first recorded MDD-related visit (index date). Following the index date, daily step counts increased gradually, but did not fully recover during the 60-day period analyzed.

To the best of our knowledge, this study is the first to show, using objective measurements, that decreased step count can be a prodromal symptom of depression. Although being able to predict the onset of depression has attracted the interest of clinicians (19, 20), information on individuals before diagnosis is lacking because most clinical studies have focused on investigating symptoms or outcomes of interventions in diagnosed patients. Prodromal symptoms are often only determined by the physician retrospectively at diagnosis. In fact, there are no available means to confirm prodromal symptoms before diagnosis. Thus, this is a breakthrough study in that it used step count data, which can be routinely obtained from workers over time, to determine change prior to diagnosis.

The results of the joinpoint regression analysis suggest a sharp decline in step counts 2 weeks prior to the first recorded MDD-related visit. This trend may be attributed to a loss of interest and motivation, which is consistent with the criteria for the diagnosis of MDD provided in the Diagnostic and Statistical Manual of Mental Disorders, fifth edition, text revision (21). Markedly diminished interest or pleasure in most activities can lead to behavioral apathy and a reduction in the daily physical activity level before MDD diagnosis. Despite the onset of depressive symptoms, Japanese people tend to wait a relatively long time before seeking medical support owing to the stigma associated with mental health issues in Japan (22). The resulting exacerbation of depressive symptoms and increased mental distress are likely to cause patients to seek help from their physicians.

This study showed a gradual recovery in daily step counts after the first visit for MDD treatment, suggesting that recovery of some physical activity occurs relatively quickly after MDD diagnosis and (presumed) treatment. However, after 60 days, the number of daily steps had not fully recovered to pre-treatment levels. The proportion of patients with MDD who achieve symptomatic remission within 4 weeks of treatment with antidepressants has been shown to be 15–25%, with an increase up to 40% observed in the following few weeks (23). Treatment efficacy may differ depending on the antidepressant drug and disease severity, however, full recovery of step counts could not be expected for patients after 60 days of treatment (24). It has been reported that it takes about a year for workers receiving antidepressants to return to previous levels of work productivity (25). Furthermore, cognitive function has been shown to be impaired even after remission and several years are needed for recovery to control levels (26, 27). In addition, workers may need an even longer period of time for full recovery of step counts if the primary mode of intervention is to reduce working hours, reduce physically demanding tasks, or to take a leave of absence after a diagnosis of depression.

An important strength of this study is the continuous collection of daily step count data from a large number of working-age individuals prior to their first visit for MDD, as well as after the diagnosis. The development of wearable devices has enabled us to monitor physical activity constantly and objectively, without any subjective biases. Our results suggest that step count data could be used to proactively detect MDD onset in the working-age population, which has the potential to lead to early interventions, and therefore, to reductions in productivity loss and the social burden imposed by MDD. Further research is needed to determine whether the approach of continuously monitoring step counts via wearable devices can be sufficiently refined to improve the rates of MDD diagnosis and to optimize subsequent interventions. Because MDD is a heterogenous disease with various physical phenotypes, a combination of methods such as step count data, other objective markers and machine learning, would be required to identify individuals at risk of MDD development in advance and to prevent the disease (28, 29).

This study has several limitations. First, recruitment was limited to individuals enrolled in employment health insurance who used a health service app. As a result, the population examined is not entirely representative of all patients with MDD who have employment health insurance or of Japanese patients with MDD in general. Second, the accuracy of the step counts recorded by the app could not be validated. Several previous studies found that step counts recorded by smartphone apps are generally reliable; however, this varies significantly depending on the app used (30–32). However, this study focused on intraindividual changes in daily step counts, and individual participants probably used the same application throughout the study period. Therefore, we believe that the effect of varying app accuracy on the results of this study was minimal. Third, the methodology did not allow us to distinguish between the first MDD diagnosis and a recurrence, or between working and non-working days, although the use of a 7-day moving average for step counts should partially address the latter limitation. Lastly, the health insurance claims data that were used in the present study did not include information about patients’ disease severity and treatment outcomes. Thus, we could not examine whether there was a correlation between step counts and severity of depressive symptoms.

In conclusion, this study found that among working-age individuals in Japan, a formal diagnosis of MDD was preceded by a notable decline in daily step counts over a period of approximately 2 weeks and was followed by a gradual increase in daily step counts after MDD diagnosis and (presumed) treatment. These results provide a foundation for further research on how to predict and prevent the development of MDD in the working-age population.

## Data availability statement

The original contributions presented in the study are included in the article/supplementary materials, further inquiries can be directed to the corresponding author.

## Ethics statement

Ethical approval was not required for the study involving humans in accordance with the local legislation and institutional requirements. Written informed consent to participate in this study was not required from the participants or the participants’ legal guardians/next of kin in accordance with the national legislation and the institutional requirements.

## Author contributions

YF, FT, and SF contributed to the study design and interpretation of study results, and were involved in the drafting, critical revision, and approval of the final version of the manuscript. All authors contributed to the article and approved the submitted version.
