# Corrigendum: Decreased step count prior to the first visit for MDD treatment: a retrospective, observational, longitudinal cohort study of continuously measured walking activity obtained from smartphones

**DOI:** 10.3389/fpubh.2024.1385467

**Published:** 2024-03-12

**Authors:** Yoshihisa Fujino, Fumie Tokuda, Shinji Fujimoto

**Affiliations:** ^1^University of Occupational and Environmental Health Japan, Fukuoka, Japan; ^2^Japan Medical Office, Takeda Pharmaceutical Company Limited, Tokyo, Japan

**Keywords:** continuous monitoring, diagnosis, Japanese, major depressive disorder, physical activity, step count, antidepressant

In the published article, there was an error. The inflection point for the joinpoint regression analysis of the 7-day moving average step counts from day −60 to day 0 was identified as occurring at day −14. However, the inflection point was actually at day −13.

A correction has been made to the Abstract, *Results*. This sentence previously stated:

“Joinpoint regression analysis of 7-day moving average step counts from day −60 to day 0 identified an inflection point at day −14.”

The corrected sentence appears below:

“Joinpoint regression analysis of 7-day moving average step counts from day −60 to day 0 identified an inflection point at day −13.”

A correction has been made to the Results, *Step counts before and after the index date (main analysis)*, Paragraph 4. This sentence previously stated:

“Joinpoint regression analysis of 7-day moving average step counts from day −60 to day 0 identified an inflection point at day −14 (Figure 3).”

The corrected sentence appears below:

“Joinpoint regression analysis of 7-day moving average step counts from day −60 to day 0 identified an inflection point at day −13 (Figure 3).”

A correction has been made to the Discussion, Paragraph 1. This sentence previously stated:

“The results show a sharp decline in daily step counts during the 14 days before the patients' first recorded MDD-related visit (index date).”

The corrected sentence appears below:

“The results show a sharp decline in daily step counts during the 13 days before the patients' first recorded MDD-related visit (index date).”

The authors apologize for these errors and state that this does not change the scientific conclusions of the article in any way. The original article has been updated.

